# Identification of Anterior Large Vessel Occlusion Stroke During the Emergency Call: Protocol for a Controlled, Nonrandomized Trial

**DOI:** 10.2196/51683

**Published:** 2024-02-13

**Authors:** Nicole Wimmesberger, Diana Rau, Florian Schuchardt, Simone Meier, Matthias L Herrmann, Ulrike Bergmann, Erik Farin-Glattacker, Jochen Brich

**Affiliations:** 1 Section Health Care Research and Rehabilitation Research, Faculty of Medicine and Medical Center University of Freiburg Freiburg im Breisgau Germany; 2 Department of Neurology and Neurophysiology Medical Centre - University of Freiburg Faculty of Medicine, University of Freiburg Freiburg Germany

**Keywords:** large vessel occlusion, emergency medical dispatch, lay first responder, emergency call, thrombectomy, stroke, thrombolysis, triage, anterior large vessel occlusion, endovascular thrombectomy, intravenous thrombolysis, modified ranking scale

## Abstract

**Background:**

Endovascular thrombectomy (ET), combined with intravenous thrombolysis if possible, is an effective treatment option for patients with stroke who have confirmed anterior large vessel occlusion (aLVO). However, ET is mainly limited to comprehensive stroke centers (CSCs), resulting in a lack of ET capacity in remote, sparsely populated areas. Most stroke networks use the “Drip and Ship” or “Mothership” strategy, resulting in either delayed ET or intravenous thrombolysis, respectively.

**Objective:**

This study protocol introduces the *Leitstellen-Basierte Erkennung von Schlaganfall-Patienten für eine Thrombektomie und daraufhin abgestimmte Optimierung der Rettungskette* (LESTOR) strategy, developed to optimize the preclinical part of the stroke chain of survival to improve the clinical outcome of patients with suspected aLVO stroke. This involves refining the dispatch strategy for identifying patients with acute aLVO stroke using a phone-based aLVO query. This includes dispatching emergency physicians and emergency medical services (EMS) to urban emergency sites, as well as dispatching helicopter EMS to remote areas. If a highly suspected aLVO is identified after a standardized aLVO score evaluation during a structured examination at the emergency scene, prompt transport to a CSC should be prioritized.

**Methods:**

The LESTOR study is a controlled, nonrandomized study implementing the LESTOR strategy, with a stepped-wedge, cluster trial design in 6 districts in southwest Germany. In an interprofessional, iterative approach, an aLVO query or dispatch protocol intended for use by dispatchers, followed by a coordinated aLVO examination score for use by EMS, is being developed, evaluated, and pretested in a simulation study. After the training of all participating health care professionals with the corresponding final aLVO query, the LESTOR strategy is being implemented stepwise. Patients otherwise receive usual stroke care in both the control and intervention groups. The primary outcome is the modified Rankin Scale at 90 days in patients with stroke receiving endovascular treatment. We will use a generalized linear mixed model for data analysis. This study is accompanied by a cost-effectiveness analysis and a qualitative process evaluation.

**Results:**

This paper describes and discusses the protocol for this controlled, nonrandomized LESTOR study. Enrollment was completed in June 2023. Data analysis is ongoing and the first results are expected to be submitted for publication in 2024. The project started in April 2020 and will end in February 2024.

**Conclusions:**

We expect that the intervention will improve the clinical outcome of patients with aLVO stroke, especially outside the catchment areas of CSCs. The results of the accompanying process evaluation and the cost-effectiveness analysis will provide further insights into the implementation process and allow for a better interpretation of the results.

**Trial Registration:**

German Clinical Trials Register DRKS00022152; https://drks.de/search/de/trial/DRKS00022152

**International Registered Report Identifier (IRRID):**

DERR1-10.2196/51683

## Introduction

### Background

Clinical outcomes of patients with acute ischemic stroke due to the occlusion of the proximal large intracranial arteries (large vessel occlusion [LVO]) remain poor when treated with intravenous thrombolysis (IVT) alone [1]. In recent years, the treatment of patients with acute LVO stroke with endovascular thrombectomy (ET), combined with IVT if possible, has been shown to produce tremendous clinical improvement [2].

In contrast to the widespread access to IVT (usually accessible at any type of stroke center—primary stroke centers [PSCs] or comprehensive stroke centers [CSCs]) in German, ET is mainly limited to CSCs due to the need for technological resources and specialized interventional physicians. This leads to a shortage of ET capacities in remote, sparsely populated areas.

Several strategies exist for the management of patients with suspected LVO in geographic areas not primarily served by a CSC, none of which are superior in terms of stroke outcome [3-6]. Currently, most stroke networks use the “Drip and Ship” (DS) or the “Mothership” (MS) strategy. According to the DS strategy, all patients with acute stroke are transferred to the nearest—in nonurban areas, mostly primary—stroke center to receive IVT if appropriate. Only if imaging indicates LVO and the patient is a candidate for thrombectomy would subsequent transfer to a CSC be initiated.

Following the MS strategy, patients with acute stroke are evaluated on scene with a stroke severity score to assess for possible LVO. Patients with a high probability of LVO stroke bypass the PSC and are transferred directly to the CSC, where both IVT and—in case of proven LVO—ET can be performed. Both the DS and MS strategies present advantages and disadvantages. The DS strategy assumes faster administration of IVT, but IVT is delayed due to the time delay in organizing and performing the transfer to the CSC [7]. On the other hand, the direct transfer to a CSC (MS strategy) results in faster initiation of ET but at the expense of delayed or even denied IVT administration due to longer ground transport times.

Since air rescue is the main means of transport for longer distances (>50 km) in the stroke network in southwest Baden-Wuerttemberg (including sparsely populated areas of the Black Forest region), optimizing the transport allocation process could shorten preclinical times in this region and comparable areas. Data from air rescue missions in Germany showed that parallel dispatch of emergency medical services (EMS) and helicopter emergency medical services (HEMS) resulted in equal arrival times of EMS and HEMS at the emergency site, shortest on-scene time, and reduced transport time to the most appropriate hospital [8]. Hence, to optimize prehospital logistics in the event of an LVO stroke, parallel dispatching of EMS and HEMS would require the identification of patients with possible LVO stroke by dispatchers in the emergency control centers (ECCs). Dispatchers already use simple stroke screening algorithms such as the “Face Arm Speech Time” (FAST) scale [9] as the basis for standardized protocol-based stroke detection in medical emergency calls. However, these simple stroke scales (relying on 1 single stroke symptom only) might be insufficient for reliable LVO detection. Lately, many publications have underlined the high value of cortical signs, such as aphasia and neglect, in combination with hemiparesis, to predict the presence of anterior LVO (aLVO), defined as emergent occlusion of the intracranial carotid artery, the tandem intracranial carotid artery, or the middle cerebral artery (M1 or proximal M2 segment) [10-12]. A recent study consistently showed that the reporting lay first responders mentioned aphasia and conjugate eye deviation (as a symptom of neglect syndrome) during emergency calls for patients with aLVO stroke [13]. It may therefore be possible to develop a stroke query specifically designed for the interaction between dispatchers and the reporting lay first responders to detect cortical signs in patients with aLVO stroke in emergency calls (“aLVO query”).

Considering the requirements and disadvantages of the DS and MS strategies, this study’s authors developed the new *Leitstellen-Basierte Erkennung von Schlaganfall-Patienten für eine Thrombektomie und daraufhin abgestimmte Optimierung der Rettungskette* (LESTOR) strategy. The aim is to optimize the dispatching strategy after the identification of patients with acute stroke with a high probability of aLVO stroke at the dispatcher level by using an aLVO query adapted to the dispatchers’ needs. This includes the dispatch of emergency physicians (EPs) in addition to EMS personnel for urban emergency sites and the parallel dispatch of an air ambulance for nonurban emergency sites for longer distances. A highly qualified EP with the most suitable rescue transport equipment arrives at the scene as quickly as possible for a structured assessment of aLVO probability by applying a standardized aLVO score. If a high suspicion of aLVO persists on-site, transport to a CSC can take place as quickly as possible.

With this strategy, this study’s authors want to eliminate the disadvantage of the delayed application of IVT in the MS strategy by enabling an early IVT administration comparable to the DS strategy with the help of the fastest possible transport, even to regions far from the CSC, while maintaining the possibility of rapid access to the MS strategy on ET.

### Objective

The LESTOR study aims to investigate whether the implementation of an aLVO query at the dispatcher level with subsequent optimization of dispatch (ie, LESTOR strategy) and a structured assessment at the emergency site improves the clinical outcomes of patients with aLVO stroke. We also compare the health-economic metrics of our approach to established allocation strategies.

### Intervention

#### Development and First Evaluation of the aLVO Query for Dispatchers

To identify patients with aLVO during telephone contact with emergency services, we intend to query the combination of hemiparesis with cortical deficits. Cortical signs (such as aphasia, neglect, and gaze deviation) in combination with hemiparesis predict the presence of aLVO with high sensitivity and specificity [10]. The Ambulance Clinical Triage for Acute Stroke Treatment (ACT-FAST) examination steps apply these fundamentals. The combination of right-sided arm paresis and aphasia or left-sided arm paresis with gaze deviation or neglect, respectively, showed high accuracy for aLVO detection in a prospective validation in EMS [14]. An advantage of the ACT-FAST algorithm is that the examination steps are adjusted to the paretic side, resulting in fewer examination steps compared to undirected scores.

The examination steps for cortical signs and the ACT-FAST algorithm serve as the starting point for the development of the aLVO query for dispatchers. This study’s authors focused on adapting the wording of the examination instructions to be comprehensible for the reporting lay first responder during the telephone query and upon collection of the results by the dispatcher on the telephone. The interprofessional development with the dispatchers of the ECCs is decisive since dispatchers are experienced in recognizing serious conditions such as cardiac arrest over the telephone. Given many years of practice in telephone resuscitation, they already have expertise in dealing with complex medical situations and communicating with laypeople who are adapted to these exceptional situations [15].

**Figure 1:**
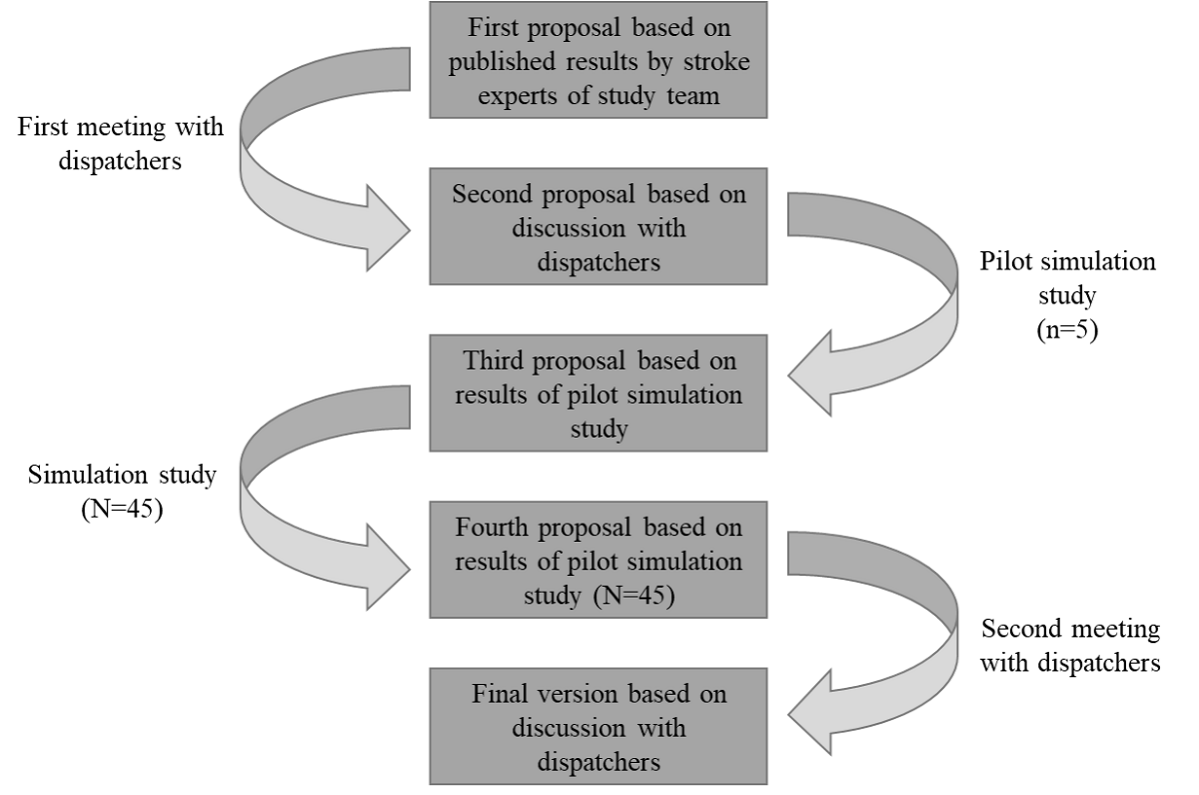
Multistage procedure in the development of the aLVO-dispatch protocol. aLVO: anterior large vessel occlusion.

Through a multistep iteration process, this study’s authors constructed an aLVO query for dispatchers aimed at identifying cortical stroke deficits (Figure 1). Stroke experts of this study’s team prepared a first proposal based on previously published results [10,16]. Dispatchers (n=6) from the ECC in Freiburg and this study’s team adjusted the wording in a consensus meeting about the specific requirements of the emergency call. We then evaluated this second proposal in a simulation study. Simulated patients simulated stroke symptoms with or without aLVO symptoms under controlled conditions. Further, 50 members of the public (potential lay first responders) were confronted with the simulated patients in a randomized block sequence. To ensure blinding, lay first responders simulating the emergency calls were positioned out of sight of dispatchers in another room. The dispatcher conducting the aLVO query with the lay first responder via telephone determined whether the simulated patient presented symptoms suspicious of an aLVO stroke. We recorded these encounters and analyzed this setting with the first participants, which served as a pilot test to check the procedure’s comprehensibility and feasibility. Subsequently, this study’s team optimized the questions’ wording, if necessary. This study’s authors then tested this third proposal with the remaining individuals (N=45).

By comparing the dispatcher’s decision with the simulated symptoms, this study’s team can determine the correct identification. We consider the aLVO query for dispatchers as sufficient if sensitivity and specificity reach at least the values that are achieved with other comparable aLVO screening scores [17] (ie, sensitivity ≥0.75 and specificity ≥0.70). Statistical case number calculations show that assuming this effect, 32 cases would be required to demonstrate significance. We therefore planned conservatively with 45 study participants (considering possible dropouts). If this study’s authors do not achieve the abovementioned aims, they will further adapt the aLVO query through more in-depth instructions.

After the simulation study, this study’s authors developed the fourth proposal of the aLVO query, which they finally discussed again with dispatchers. The dispatchers and this study’s team approved a final version of the aLVO query for dispatchers. By using this complex, multistage procedure, we are striving for the best possible combination of test quality criteria, practical feasibility, and a high level of acceptance during the implementation phase.

#### Development of the aLVO-Dispatch Protocol and Integration in the Stroke Detection Protocols in the ECC

We aim at establishing an aLVO-dispatch protocol that queries for eligibility for thrombectomy if the aLVO query is positive. Therefore, the aLVO-dispatch protocol consists of the final aLVO query, which, if aLVO is suspected, is expanded to include a query on the patient’s previous state of health and the time window in which the stroke symptoms occurred. The aLVO-dispatch protocol is “LESTOR positive” at the ECC level only, if aLVO is suspected in patients who are not bedridden within the 24-hour window.

All the participating ECCs use electronic dispatch systems with protocol-based dispatching programs. For stroke detection in general, the FAST algorithm is used in all participating ECCs, followed by the immediate dispatch of EMS. We maintain this procedure and implement our aLVO-dispatch protocol directly after completing the first dispatch. Immediately afterward, the dispatcher carries out the aLVO query. If the aLVO-dispatch protocol results in “LESTOR positive,” the dispatchers check whether they can optimize the dispatching strategy: within the catchment areas of the CSC, a ground-based EP is dispatched in addition to EMS; outside the catchment area of the CSC, a physician-staffed helicopter is dispatched in parallel. Both protocol-based dispatching programs (FAST and aLVO query) are not mandatory, as the dispatcher can dispatch for unambiguous cases, bypassing the structured dispatching programs to perform faster dispatching.

#### Development of the aLVO Query for EMS or HEMS

Since EPs and EMS or HEMS need to confirm the aLVO stroke suspicion established by the dispatchers after arrival on the scene, an analogous 2-step procedure is implemented as a secondary survey on scene. In the first step, they apply the FAST scale. If it is positive, they examine cortical symptoms indicative of an aLVO stroke. To enable a comparison between the results of the ECC and EMS or EP or HEMS, as well as to establish a “common” and concerted language at the ECC and EMS or HEMS interface, we closely adapted the examination steps of the aLVO query used in the ECC to the use in EMS or EP or HEMS (“LESTOR Score”).

#### Development of Seminars for Training or Implementation

##### General Structure of the Seminars

Since the intervention involves neurological examination steps of the patient with stroke that were not previously carried out by the dispatchers or the EMS, EP, or HEMS personnel, this study’s authors developed training seminars for all professionals involved in the project. We used didactically carefully designed units for knowledge transfer, including interactive, case-based examples. The didactic training concept builds on principles of the “adult learning theory” (eg, linking to everyday needs and teaching problem-oriented approaches) [18]. Experienced stroke physicians who are part of our study team taught stroke symptoms, recanalizing therapies, and existing referral concepts (DS and MS) with their potential barriers. This study’s team then introduced the LESTOR strategy as a new referral concept assessed in this study. aLVO detection, the prerequisite of this strategy, is visualized with the use of video samples demonstrating simulated patients with hemiparesis and the corresponding cortical symptoms. The stroke experts also taught practical applications of the examination steps and their interpretation, including pitfalls. Due to the COVID-19 pandemic, all the training was held digitally using videoconferencing software. To enable discussions, time slots for discussions were planned at 3 predetermined time points within each seminar.

##### Adaptions of Seminars for Dispatcher Training

Due to limited practical familiarity with strokes, adjustments for dispatchers included a more detailed description of stroke symptoms, therapies, and referral concepts. Adapted to the dispatchers’ workplace situation, we taught the content and application of the aLVO query additionally with a recorded simulated emergency call. To optimize training success, small groups of 4 dispatchers were taught in 4-hour sessions, resulting in 6 to 10 training seminars for each ECC.

##### Adaptions of Seminars for EMS or HEMS Personnel and EP Training

In the 2-hour seminars for EMS or HEMS personnel and EPs, we introduced the “LESTOR Score” that complements the examination of patients with FAST-positive stroke at the emergency site. To this end, the specifically designed LESTOR app offers additional teaching of the examination steps and algorithm support (see below). Special focus lies on the allocation and prenotification process for patient who are LESTOR positive s.

#### Supporting Materials

##### LESTOR App

This study’s team has developed a freely available mobile app intended to support the implementation and penetration of the “LESTOR Score” in the EMS or HEMS and for EPs. The main aim of the app is to consolidate knowledge through e-learning modules (eg, instructions, illustrations, and explanatory videos).

##### Further Materials

Professionals participating in this study can access training and study content digitally to repeat content and train new personnel in the LESTOR approach, including pocket cards and handouts for EPs and EMS or HEMS personnel.

#### Intervention Implementation

The intervention consists of (1) the implementation of the aLVO-dispatch protocol for suspected stroke at the ECC level, (2) the adjustment of the dispatch in case of a positive aLVO query result, (3) the structured aLVO reassessment on-scene, and (4) the referral strategy of patients with stroke depending on the aLVO probability.

First, after accomplishing stroke dispatch following a positive FAST score, the aLVO-dispatch protocol is started automatically in the ECC, resulting in either “LESTOR negative” (with no further action) or “LESTOR positive” results.

Second, a “LESTOR positive” result of the aLVO-dispatch protocol necessitates a review of the dispatch made: inside the catchment areas of the 2 CSCs, a ground-based EP is dispatched in addition to EMS, whereas outside the catchment area of the 2 CSCs, HEMS is dispatched in parallel.

Third, on the scene, in case of a FAST-positive stroke, EMS, HEMS, or EPs repeat the aLVO query, by applying the aLVO query customized for their situation (“LESTOR Score”), supported by the mobile app or the pocket card.

Finally, the referral strategy of patients with stroke depends on the result of the LESTOR Score on the scene, time window, and preexisting severe impairment due to another disease.

Patients who are “LESTOR positive” without severe impairment due to another disease, and where the duration of symptoms is <24 hours, are transported to the next CSC: via ground-based dispatch when inside the catchment areas of the CSCs, and via air-based dispatch when outside the catchment area of the 2 CSCs. All other patients with stroke are transported to the nearest stroke unit, regardless of whether they are a PSC or CSC.

## Methods

### Overview

The LESTOR study comprises various process stages and an accompanying process evaluation. We prospectively registered the trial for the World Health Organization Universal Trial Number and in the German Clinical Trials Register (DRKS00022152) on October 19, 2020.

### Study Design

**Figure 2:**
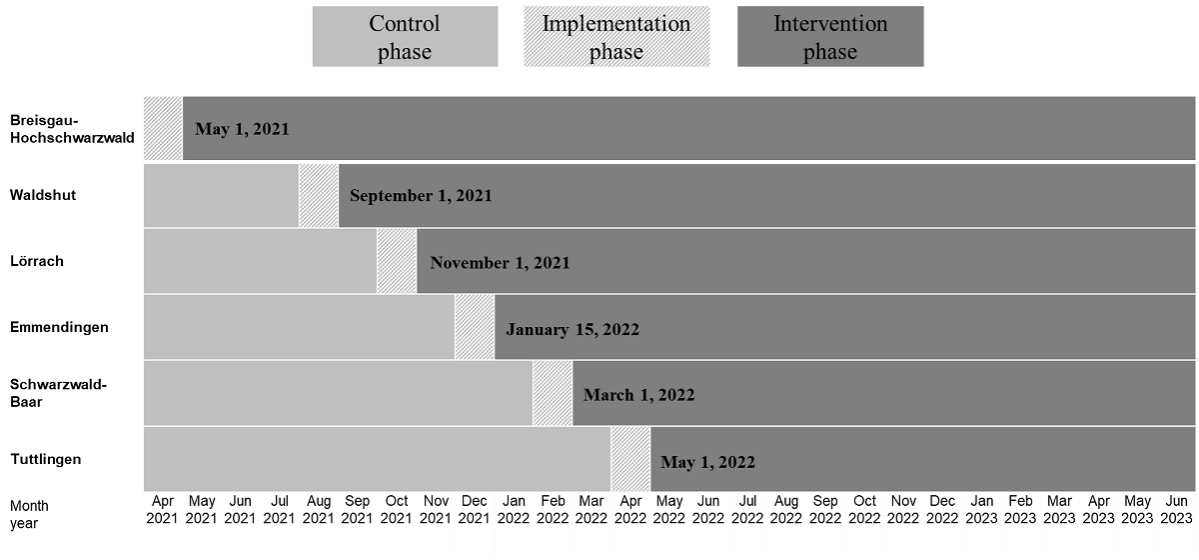
Stepped wedge, cluster trial design of the LESTOR study in 6 districts. LESTOR: Leitstellen-Basierte Erkennung von Schlaganfall-Patienten für eine Thrombektomie und daraufhin abgestimmte Optimierung der Rettungskette.

This study’s authors performed a controlled, nonrandomized study, introducing the LESTOR strategy with a nonrandomized, stepped wedge, cluster trial design in 6 districts (ie, clusters) in Baden Wuerttemberg in southwest Germany (Figure 2).

Each cluster includes 1 ECC. Ground emergency medical ambulances are nearly equally distributed across the districts. All 6 districts are supported by 3 air rescue facilities. Further, 6 PSCs and 2 CSCs provide stroke service. The research team sequentially assigns the clusters to the intervention at intervals of 2 to 3 months in an unblinded manner (Figure 2).

This study’s team implemented the multicomponent LESTOR approach simultaneously for each cluster’s ECC, EMS personnel, EPs, and stroke units. Since HEMS were involved in the intervention from the beginning, this study’s authors introduced the LESTOR approach to the 3 air rescue facilities before the implementation of the first cluster.

Each cluster passed through the following phases:

Phase 1 was the control phase (preintervention baseline phase, 3-15 months). During phase 1, ECCs carried out the emergency call and stroke dispatch by local standards: they used the FAST scale for stroke detection, followed by the immediate dispatch of EMS (with additional EP dispatch depending on local conditions). EMS personnel and EPs also used the FAST scale for stroke detection at the scene. The standard allocation for stroke patients was the nearest stroke unit (regardless of whether it is a PSC or CSC). Allocation to CSCs outside their catchment area was based on an individual decision by the EMS or EP to contact the CSC and requires CSC approval. If a helicopter was considered to be the most suitable means of transport on-site, a sequential additional request for the helicopter must be made.

Phase 2 was the implementation phase, when all the professionals involved in acute stroke patient care in a cluster participated in seminars (see above) paralleling the technical implementation of the aLVO query.

Phase 3 was the intervention phase (14-26 months), which follows the intervention’s implementation as described above.

### Patient Recruitment

In total, 6 districts in Baden Wuerttemberg in southwest Germany participated in the LESTOR study (Breisgau-Hochschwarzwald, Loerrach, Emmendingen, Schwarzwald-Baar, Tuttlingen, and Waldshut) including 6 ECCs; the EMS and EPs of all districts; 3 air rescue facilities; and all the stroke centers of the 6 districts: 2 CSCs (Freiburg im Breisgau and Villingen-Schwenningen) and 6 PSCs—5 of them having a telestroke connection. The maximum distances to CSCs in this study’s area were about 120 km by ground and about 80 km by air, while the distances to PSCs were mostly less than 30 km by ground. The distribution of stroke units was specified by the Ministry of Social Affairs in Baden Wuerttemberg, Germany [19].

The 6 districts cover a population of about 1.4 million people living on almost 6000 square kilometers; population density in this area is 233 per square kilometer on average, with the minimum in the district Waldshut (151.4 per square km) and the maximum in the district of Loerrach (283.7 per square km) [20]. According to the Organisation for Economic Co-Operation and Development “Urban-rural classification for NUTS 3 regions” [21], all 6 districts are classified as intermediate regions (rural population between 20% and 50% of the total population). As a regional peculiarity, 5 out of 6 districts have a share of the sparsely populated Black Forest with areas that are difficult to access.

Each district had 1 ECC, operated by the aid organization “Red Cross” and the state administration of each district. EMS was provided in the vast majority by the aid organizations “Deutsches Rotes Kreuz (Red Cross)” and “Malteser,” while EPs were organized by the hospitals assuring EP coverage within each district. EMS and EPs were organized at the district level. All 6 districts were supported by 3 air rescue facilities (the German *Deutsche Rettungsflugwacht e. V. Luftrettung*, the Swiss *Rettungsflugwacht Garde Aérienne*, and the Swiss Alpine Air Ambulance) with 5 helicopters during the day and 4 helicopters at night.

### Inclusion and Exclusion Criteria

#### Inclusion Criteria

Eligible for inclusion were patients aged older than 18 years presenting stroke symptoms that can be treated within 24 hours of the initial symptoms’ onset.

#### Exclusion Criteria

Exclusion criteria were preexisting severe impairment due to another disease (corresponding to level 5 of the modified Rankin Scale [mRS]: “Severe disability; Bedridden, incontinent, requires constant nursing assistance”) [22-24], patients in a coma (National Institutes of Health Stroke Scale [25] item of consciousness>2), patients with an unstable clinical status who required emergent life support care, patients hospitalized with acute stroke and suspected aLVO, and patients unlikely to be available for the 90-day follow-up (eg, no fixed home address or visitor from overseas).

### Primary and Secondary Outcomes

#### Primary Outcome

The primary outcome was the mRS score at 90 days in patients with ischemic stroke who received ET. The mRS measures the degree of disability after a stroke with a score ranging from 0 to 6 (0=no deficit; 1=minor deficit without limitations; 2=deficit with limitations; 3=dependence, but independent walking possible; 4=dependence, independent walking no longer possible; 5=being bedridden; and 6=death) [22-24]. The primary outcome was assessed by a structured telephone interview conducted by a central assessor blinded to group allocation.

#### Secondary Outcomes

Prespecified secondary outcomes were the dichotomization of the mRS (day 90) into scores of 0-2 and 3-6 and the time from emergency call to hospital admission.

### Control Variables

Control variables were age, sex, pre-mRS score, and severity of stroke (National Institutes of Health Stroke Scale) [25] at hospital admission.

### Data Collection

This study’s team asked each participating ECC to share their aLVO-query results with the research team along with time metrics. This study’s authors also collected deidentified case data from the 2 ECCs receiving endovascular treatment in a database. A study staff member blinded to group allocation requested the 7-point (0-6) mRS by telephone from patients with stroke receiving endovascular treatment or their guardians 90 days after stroke (unblinded treatment and blinded end point assessment). For the health economic evaluation, this study’s authors asked the medical controlling department of the University Medical Centre of Freiburg.

### Health Economic Evaluation

This study’s team will analyze the costs associated with hospitalization and the intervention for all the patients. Data will be provided by the medical controlling department of the University Medical Centre. This study’s authors will also perform a cost-effectiveness analysis, whereby the primary end point (mRS) is related to the total costs.

### Power Considerations

A power analysis showed that testing for the statistically significant superiority of the intervention over the control group required far more cases (N=664) than available in the target region during this study’s period. This study’s authors, therefore, conducted a controlled feasibility study to explore achievable effects and practicability under routine care conditions. This study’s team will explore the clinical significance of the intervention effects and refrain from testing for the statistical superiority of the intervention over the control group.

### Statistical Analysis

We will use a generalized linear mixed model following the basic model proposed by Hussey and Hughes [26], including the 6 districts as clusters. This study’s team will extend the model as proposed by Hemming et al [27] and Li et al [28] to control for the secular trend to avoid potential bias. We will therefore stratify the sample by the 2 stroke centers and add a fixed effect for the secular trend stratified by each of these 2 hospitals, taking into account the control variables. We will assume the same treatment effect in both strata but will exploratively test this assumption by extending the model with an interaction term.

As studies have shown that it is advantageous to examine the full-scale range of mRS—rather than dichotomizing it—this study’s authors will account for the ordinal scaling of the mRS [29].

We will conduct exploratory subgroup analyses for cases within and outside of the catchment areas of the 2 CSCs and consider the dichotomous mRS because a favorable clinical outcome is defined as mRS≤2 similar to Goyal et al [2]. In addition, the area under the curve for different mRS cutoff values will be calculated in an exploratory sensitivity analysis.

### Missing Values

We will use multiple imputations [30], assuming that the data are missing at random, and perform a full case analysis as a sensitivity analysis for the imputed model.

### Process Evaluation

The process evaluation will consist of semistructured, guideline-based individual telephone interviews conducted by trained researchers to assess the implementation process at 2 points in time (in the middle of and at the end of the intervention phase). This study’s authors will interview representatives of the ECC, EMS and HEMS personnel, EPs, and first responders. A study nurse will recruit lay first responders of patients receiving ET at the University Hospital of Freiburg to participate in interviews. Key topics for the interviews with medical professionals will be the feasibility of implementation, accessibility, acceptability, appropriateness, fidelity, penetration, and sustainability of the intervention. This study’s authors will ask lay first responders about the emergency and personal stress, and the instructions given during the emergency call. Further, 2 independent researchers will use Kuckartz’s [31] multistage qualitative content analysis to analyze transcribed data using MAXQDA (VERBI Software GmbH) software.

The results of the first interview round will provide input for a midintervention workshop for all participating medical professionals involved in this study. The workshop aims to discuss the experience and data gained to improve the intervention. The results of the second interview round may help to optimize the intervention for future implementations and projects.

### Ethical Considerations

This study’s authors did not expect any disadvantages for patients when applying the aLVO query. Even if the aLVO assessment failed and aLVO-positive cases were missed, patients received standard care.

By the local ethics committee, this study’s team had established a waiver of consent approach, as a high proportion of patients with stroke with aLVO experienced severe neurological sequelae and had diminished capacity to consent. Moreover, acute psychological distress occurred in approximately 30% of cases during the acute phase, and obtaining consent from relatives, legal guardians, or health care proxies was only possible in a small proportion of cases without causing undue additional psychosocial stress for the representatives [32]. The researchers inform patients using an invitation about a telephone call, which addresses the patient’s further neurological course at 90 days after stroke.

For the process evaluation, the interview partners will be informed, in written and verbal form, about this study and must have agreed to the procedure. Project participation will be voluntary and consent can be revoked at any time.

Ethical approval for this study was obtained from the Ethics Committee at the University of Freiburg (416/20; August 10, 2020). The project started in April 2020 and will end in February 2024. All procedures were performed in accordance both with the ethical standards of the institutional or national research committee, and the 1964 Helsinki Declaration and its later amendments or with comparable ethical standards. This study protocol adheres to the recommended SPIRIT (Standard Protocol Items: Recommendations for Interventional Trials) checklist.

## Results

The development phase of this study was completed and the successful integration of the LESTOR strategy into the daily work was in place before patient enrollment. This study was conducted between April 2020 and February 2024, with final data collected by June 2023 and final analyses and results anticipated by mid-2024.

## Discussion

### Relevance

More than 7 years after the establishment of ET as the standard therapy for aLVO stroke, the best prehospital care and strategy for patients with aLVO stroke, particularly outside the catchment areas of CSCs, remains unclear.

This study aims to evaluate a new strategy, targeting the essential steps of the prehospital section of the stroke chain of survival. The detection of aLVO stroke in the emergency call enables optimization of the dispatch process in terms of human expertise as well as for means of rescue. Involving the lay first responder in the clinical examination of cortical signs for aLVO detection over the telephone represents a major challenge, which this study’s authors aim to address with their multistage, interprofessional development process for the aLVO query in the ECC. Re-establishing the aLVO suspicion by EMS, HEMS, or EPs necessitates the introduction of a structured aLVO examination, which the stroke study team closely adapted to the examination steps of the aLVO query used in the ECC (“LESTOR Score”). The LESTOR strategy might enable a delay-free transport to the CSC and could therefore overcome the disadvantage of the MS strategy and still provide an equally fast initiation to thrombolysis as the DS strategy by maintaining the faster access to ET.

As this new strategy does not require any additional human or material resources, other than training time for all the parties involved, it may be easy to implement in comparable regions. The extensive accompanying process evaluation and the health economic evaluation should help to identify determinants of the implementation and thus optimize the transferability.

### Limitations

The project faces several challenges. The pieces of training need to reach many dispatchers, EMS and HEMS personnel, and EPs in all 6 districts. Due to the COVID-19 pandemic, the pieces of training were delivered digitally with videoconferencing software. While this enables more flexibility in terms of participation, it may reduce interactions and learning compared to face-to-face sessions. Despite the extensive development of the query, certain known problems with the detection of a stroke in an emergency call cannot be solved, for instance, the misjudgment of strokes as falls or misclassification of aphasia as confusion or reduced vigilance. Moreover, not all callers may be able to understand and perform the instructions for the aLVO query due to stress during the emergency situation or language barriers. Although this study’s team plans the trial in an area with 1.4 million inhabitants, the expected number of patients with aLVO stroke during this study’s phase is likely to be too small to assess the statistical superiority of the intervention and could therefore be addressed in future trials.

### Conclusion

The LESTOR study aims to optimize prehospital care for patients with aLVO stroke with a focus on reducing inequality in aLVO stroke care within and outside of CSC catchment areas. The proposed LESTOR strategy addresses the earliest possible detection of aLVO symptoms in patients suspected of having a stroke, by using the results of the LESTOR query in the emergency call to guide dispatch decisions. This may enable optimization of the dispatch in terms of human expertise as well as for means of rescue and, in case of aLVO suspicion, create the conditions for a fast and direct transport to the CSC, including the immediate use of air rescue for longer distances to the CSC. The results of the accompanying process evaluation and the cost-effectiveness analysis will provide further insights into the implementation process and allow for a better interpretation of the results.

## Abbreviations

ACT-FAST: Ambulance Clinical Triage for Acute Stroke Treatment
aLVO: anterior large vessel occlusion
CSC: comprehensive stroke center
DS: Drip and Ship
ECC: emergency control center
EMS: emergency medical services
EP: emergency physician
ET: endovascular thrombectomy
FAST: Face Arm Speech Time
HEMS: helicopter emergency medical services
IVT: intravenous thrombolysis
LESTOR: Leitstellen-Basierte Erkennung von Schlaganfall-Patienten für eine Thrombektomie und daraufhin abgestimmte Optimierung der Rettungskette
LVO: large vessel occlusion
mRS: modified Rankin Scale
MS: Mothership
PSC: primary stroke center
SPIRIT: Standard Protocol Items: Recommendations for Interventional Trials

## Data Availability

Data sharing is not applicable to this work as no data sets were generated or analyzed.
