# Correction: Identification of Anterior Large Vessel Occlusion Stroke During the Emergency Call: Protocol for a Controlled, Nonrandomized Trial

**DOI:** 10.2196/58180

**Published:** 2024-04-16

**Authors:** Nicole Wimmesberger, Diana Rau, Florian Schuchardt, Simone Meier, Matthias L Herrmann, Ulrike Bergmann, Erik Farin-Glattacker, Jochen Brich

**Affiliations:** 1 Section Health Care Research and Rehabilitation Research, Faculty of Medicine and Medical Center University of Freiburg Freiburg im Breisgau Germany; 2 Department of Neurology and Neurophysiology Medical Centre - University of Freiburg Faculty of Medicine, University of Freiburg Freiburg Germany

In “Identification of Anterior Large Vessel Occlusion Stroke During the Emergency Call: Protocol for a Controlled, Nonrandomized Trial” (JMIR Res Protoc 2024;13:e51683) the authors noted the following error.

In the originally published manuscript, the following authors’ degrees were incorrectly listed as:

Florian Schuchardt, PhD

Simone Meier, PhD

Matthias L Herrmann, PhD

Jochen Brich, PD Dr

This has been corrected to:

Florian Schuchardt, MD

Simone Meier, MD

Matthias L Herrmann, MD

Jochen Brich, MD

The correction will appear in the online version of the paper on the JMIR Publications website on April 16, 2024, together with the publication of this correction notice. Because this was made after submission to PubMed, PubMed Central, and other full-text repositories, the corrected article has also been resubmitted to those repositories.

